# Bounding uncertainty in volumetric geometric models for terrestrial lidar observations of ecosystems

**DOI:** 10.1098/rsfs.2017.0043

**Published:** 2018-02-16

**Authors:** Ian Paynter, Daniel Genest, Francesco Peri, Crystal Schaaf

**Affiliations:** School for the Environment, University of Massachusetts Boston, Boston, MA, USA

**Keywords:** lidar, forestry, erosion, geometry, modelling

## Abstract

Volumetric models with known biases are shown to provide bounds for the uncertainty in estimations of volume for ecologically interesting objects, observed with a terrestrial laser scanner (TLS) instrument. Bounding cuboids, three-dimensional convex hull polygons, voxels, the Outer Hull Model and Square Based Columns (SBCs) are considered for their ability to estimate the volume of temperate and tropical trees, as well as geomorphological features such as bluffs and saltmarsh creeks. For temperate trees, supplementary geometric models are evaluated for their ability to bound the uncertainty in cylinder-based reconstructions, finding that coarser volumetric methods do not currently constrain volume meaningfully, but may be helpful with further refinement, or in hybridized models. Three-dimensional convex hull polygons consistently overestimate object volume, and SBCs consistently underestimate volume. Voxel estimations vary in their bias, due to the point density of the TLS data, and occlusion, particularly in trees. The response of the models to parametrization is analysed, observing unexpected trends in the SBC estimates for the drumlin dataset. Establishing that this result is due to the resolution of the TLS observations being insufficient to support the resolution of the geometric model, it is suggested that geometric models with predictable outcomes can also highlight data quality issues when they produce illogical results.

## 1. Introduction

This study investigates whether methods with guaranteed directions of bias can constrain the uncertainty in volume estimates of ecosystem objects observed by terrestrial laser scanner (TLS) data. TLSs are light detection and ranging (lidar) instruments [1] that are either mobile [2,3], or mounted on ground-based platforms such as tripods [4–8], towers [9] and all-terrain vehicles [10,11]. Lidar data have been used to retrieve many important ecosystem properties [12–14], including tree volume for carbon storage estimation [15–19]; canopy structure for radiative transfer [20–22]; aspects of geomorphological form, such as the width and depth of channels, for hydrological modelling [23]; and geomorphological change for coastal management [24].

Retrievals of ecosystem structural properties from lidar data, particularly from TLS instruments, are often achieved through modelling approaches, referred to henceforth as geometric models, that reconstruct approximations of the geometry of objects in the ecosystem from the discrete and discontinuous observations of structure made by the TLS lidar. For example, the detailed reconstruction of tree structure from TLS data uses quantitative structure models to form a hierarchical, connected network of cylinders [25]. These cylinder models permit estimates of woody volume, linked to biomass estimates with allometric equations for wood density [18,26,27].

While geometric models for use with TLS data have become increasingly refined, the uncertainty in the resulting estimations of ecosystem properties can only be calculated as error, validated with direct measurement of the ecosystem properties. For example, uncertainties in TLS observations of woody volume are established by destructive sampling of observed trees [26–28]. However, validating every TLS observation with direct measurements of the ecosystem properties of interest would be pointless. It is possible to provide some inference about the quality of the representation of an object in a set of TLS observations by examining the stability of geometric models by varying their parametrization, or with repeats, if the models include a random element. However, there is no guarantee that the true value of the property of interest lies within the observed range of variation. Therefore, estimates of uncertainty established by direct validation for a particular instrument, for a particular target, under particular conditions, will always have questionable scope of inference, especially for ecosystem objects with highly variable morphology, such as trees. Thus, it would be highly desirable to retrieve valid estimates of uncertainty from within a given TLS observation, rather than propagating them from previously validated observations.

Geometric models that have a predictable interaction with object geometry, and therefore have a known directional bias of error, could provide uncertainty bounds for individual TLS observations. In other words, methods whose estimates can be assumed to be underestimations could provide lower bounds to volume estimates, and methods whose estimates are assumed to be overestimations could provide upper bounds. An example would be a three-dimensional convex hull polygon (3D CHP) [29], which encompasses a set of points without forming any concave features. These constraints ensure the volume of the resulting polygon to be an overestimation of the volume of the object described by the points, and therefore a definite upper bound on the estimate of volume.

Since many of the geometric properties of objects observed with TLS become inputs to larger scale ecological modelling efforts, such woody volume for carbon storage monitoring [30], or geomorphological influences on erosion rates [24], constraining the uncertainty for these inputs would help ensure that the outputs of those ecological models can be relied upon for decision-making and management. Furthermore, providing meaningful constraints on ecological properties should be useful to validate coarser resolution, but larger extent estimates of ecosystem properties, such as those derived from satellite resources. In particular, space-based remote-sensing observation of structure, such as from the forthcoming Global Ecosystem Dynamics Investigation (GEDI) mission [31] could benefit from such structurally explicit evaluation tools.

This study explores the ability of geometric models to bound the uncertainty in volume estimates for a variety of ecological objects from various ecosystems, and of various forms, represented in TLS observations. Several species of temperate tree, *Ceiba pentandra* tree buttressed roots, an old-growth *Ficus aurea* (strangler fig), the coastal bluff formed by an eroding drumlin and a saltmarsh creek are all represented by examples in this study. These highlight different opportunities and challenges, from the simple continuous surface of a drumlin, observable from an unobstructed viewpoint, to the convoluted structure of trees, which have large overall extents with relatively small, but complexly arranged volumes.

The volumetric estimation methods evaluated in this study include the Bounding Cuboid [32], 3D CHP [29], Square Based Column (SBC) and the Outer Hull Model (OHM) [27]. For estimating the volume of temperate trees, volume estimation methods are assessed for their ability to provide uncertainty bounds to supplement the cylinder-based quantitative structure model [25].

## 2. Material and methods

### 2.1. Terrestrial laser scanner instruments

**Figure 1.**
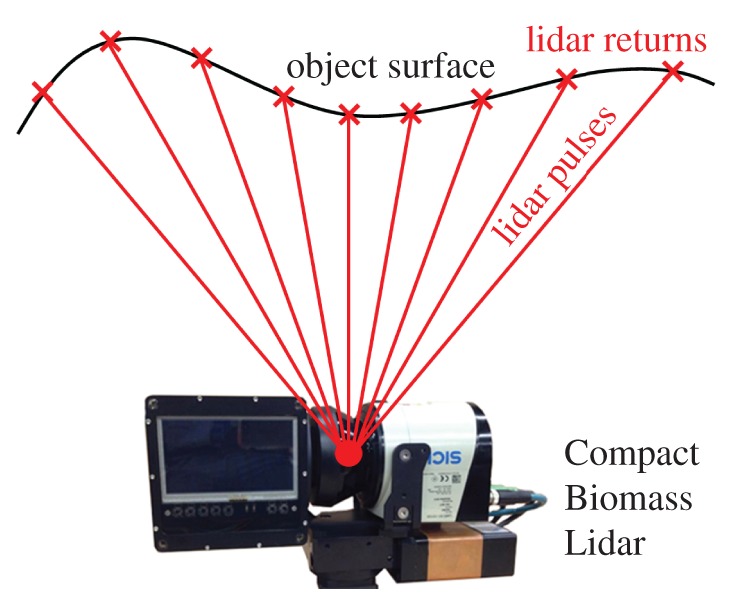
Diagram of lidar pulses (red lines) sampling a hypothetical surface (black line), featuring an image of the Compact Biomass Lidar (CBL2) instrument. Note the discontinuous sampling of the surface and the range-dependency of the density of lidar returns along the object's surface. (Online version in colour.)

We make use of TLS datasets acquired using the first and second iterations of the University of Massachusetts Boston Compact Biomass Lidar (CBL1 and CBL2, figure 1, also described in [9]). These 905 nm, discrete, time-of-flight instruments have moderate sampling resolution (0.25° horizontal, 0.25°/0.5° vertical for CBL1 and CBL2, respectively) and maximum range (approx. 40 m). However, these instruments were used for these exploratory studies due to the deployment flexibility lent by their light weight (3.5 kg) and rapid scan acquisition (33 s at 20 kHz pulse rate, of pulses with first and second returns).

### 2.2. Data processing

For a variety of target objects of ecological interest, multiple consecutive scans were obtained, either with CBL1 or CBL2 (table 1 for details). The point clouds, derived from these scans as per [9], were co-aligned (adjusted for relative scanning position and orientation) using automatic approaches where possible. Final co-alignments were adjusted manually in point cloud visualization software by a single operator.

**Table 1.** Description of Compact Biomass Lidar (CBL1 and CBL2) datasets, including scanning configurations and method applications.

dataset	instrument	no. scans	year	location
*Ceiba pentandra* 1	CBL1	8	2014	La Selva Biological Station, Sarapiqui, Costa Rica
*Ceiba pentandra* 2	CBL2	8	2017	Jardin Botanico UPR, San Juan, Puerto Rico, USA
*Ceiba pentandra* 3	CBL2	6	2017	Jardin Botanico UPR, San Juan, Puerto Rico, USA
temperate urban trees (×12)	CBL2	4	2015, 2016	Boston, MA, USA
eroding drumlin bluff	CBL1/CBL2	3	2014, 2015	Lovell's Island, MA, USA
*Ficus aurea*	CBL1	30	2014	Corcovado, Costa Rica
saltmarsh creek	CBL2	4	2016	Plum Island LTER, MA, USA

### 2.3. Datasets

The datasets included in this study comprise the buttress-rooted bases of several *Ceiba pentandra* trees (La Selva Biological Station, Sarapiqui, Costa Rica and Jardin Botanico UPR, San Juan, Puerto Rico); a large, free-standing *Ficus aurea* (strangler fig) tree, approximately 45 m in height, and with no remaining structure from the original host tree (Corcovado, Costa Rica); a saltmarsh creek (Plum Island LTER, MA, USA); a bluff formed from an eroding drumlin, observed in both 2014 and 2015 (Lovell's Island, MA, USA); and 12, individually scanned, temperate trees in managed urban environments (5 × *Acer platanoides* (Norway maple), 3 × *Acer rubrum* (red maple), 3 × *Quercus rubra* (red oak), 1 × *Robinia pseudoacacia* (black locust) (Boston, MA, USA)). For full details, see table 1.

### 2.4. Volume estimation methods

Several geometric models for estimating the volume of objects of ecological interest were used in this study. These methods are described below, and their application to the various datasets, as well as their expected biases, are documented in table 2.

**Table 2.** Results from the Bounding Cuboid, three-dimensional convex hull polygons (3D CHP), Outer Hull Model (OHM), voxels and Square Based Column (SBC) of estimating volume for the objects analysed in this study. All volumes are in cubic metres.

method of volume estimation	expected bias	object				
		*C. pentandra* 1	*C. pentandra* 2	*C. pentandra* 3	*F. aurea*	saltmarsh creek
Bounding Cuboid	overestimate	291.28	274.35	169.17	111984.76	1445.88
3D CHP	overestimate	111.55	68.08	45.14	44 748	881.89
OHM (max.)	overestimate	71.275	50.28	31.34	24693.43	857.25
OHM (min.)	overestimate	65.32	21.92	20.10	20452.94	769.66
voxels (max.)	unknown	122	86	50	4087.88	—
voxels (min.)	unknown	34.01	6.82	4.98	581.06	—
SBC (max.)	underestimate	27.11	14.70	7.93	—	631.81
SBC (min.)	underestimate	19.38	3.32	3.25	—	615.08

**Figure 2.**
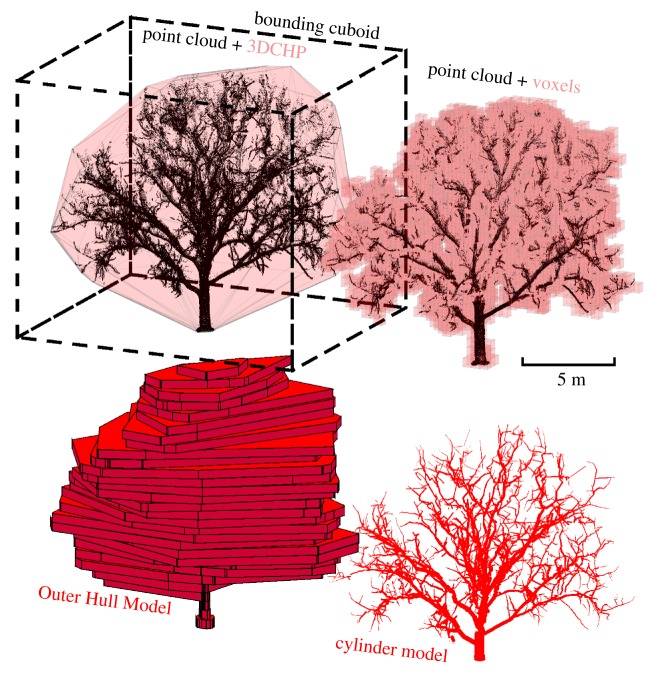
Visualizations of the geometric models applied to the *Robinia pseudoacacia* tree. Top left: point cloud, three-dimensional convex hull polygon (3D CHP) and Bounding Cuboid (dashed lines). Top right: voxels (0.5 voxel size). Bottom left: Outer Hull Model (OHM, 0.5 m bin height). Bottom right: cylinder-based quantitative structure model [25]. (Online version in colour.)

The *Bounding Cuboid* is defined as the smallest cuboid that can fully encompass a point cloud representation of an object [32], given rotation only about the *Z*-axis. The 3D CHP is the shape of smallest volume, consisting of connected polygons, and with no concave features, that can encompass a set of points [29]. *Voxelization* establishes equally sized cubes arranged on a local grid to describe a point cloud [33,34]. In this study, voxels are established in response to the presence of one or more returns in the space enclosed by the voxel. For the datasets representing complete trees, we apply a quantitative structure model developed by Raumonen *et al.* [25] that describes TLS representations of trees as a connected network of cylinders. This model was parametrized based on prior investigations (PatchDiam1 = 0.20; BallRad1 = 0.10; nmin1 = 15; PatchDiam2 = 0.07; BallRad2 = 0.08; nmin2 = 10; lcyl = 5). Visualizations of the methods for the temperate trees can be seen in figure 2.

The OHM [27] consists of a series of convex hull polygons. Each convex hull polygon in the OHM methods is fitted to two-dimensional Euclidean coordinates of a subset of a point cloud, delineated by binning the points into sections of consistent thickness. Multiplying the sum of the area of these convex hull polygons by the thickness of the bins therefore produces an estimate of the total volume of an object. In this study, OHM is exclusively employed with the convex hull polygons stacked in the *Z*-axis (therefore, delineated by height).

The SBC method involves the projection of columns of equal-sized square profile from a grid arranged on a two-dimensional plane, until they encounter a point in a representation of an object. If a point is not encountered, then a column is not established. The plane is arranged based on the typical geometry of the archetype of object being represented, such that the largest proportion of the volume of the object, and only the volume of the object (rather than the space around it) is expected to be represented. The arrangement of the SBC plane can be thought of, and potentially solved, as a maximization problem for the volume of the object, but a good rule of thumb is that the geometry of the object should be predominantly concave relative to the viewpoint of the plane. For example, the plane for SBC is established across the top of the saltmarsh creek, and directly beneath the bases of trees with buttressed roots. For the analysis of the eroding drumlin in this study, the volume established with SBC describes the relative space between the plane and the surface of the bluff in 2014 and 2015, and therefore the change resulting from erosion.

The influence of the parametrization of several of the geometric models on their estimation of volume was of interest in this study. Therefore, for each object of ecological interest, we implemented a range of *voxel size*, *grid sizes* for SBC and *bin heights* for OHM, as described in table 2.

## 3. Results

**Figure 3.**
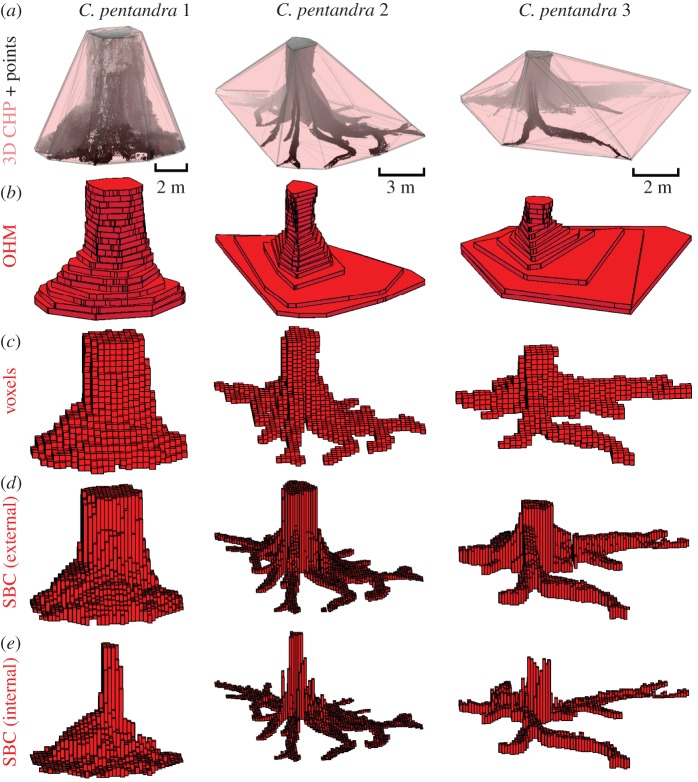
Visualizations of volume estimation methods for *Ceiba pentandra* trees 1, 2 and 3 (left, middle, right columns, respectively). (*a*) *Ceiba* tree point clouds with three-dimensional convex hull polygons (3D CHP); (*b*) Outer Hull Model (OHM, 0.3 m bin height); (*c*) voxels (0.3 m voxel size); (*d*) Square Based Column (SBC) using maximum height (0.3 m grid size) and (*e*) SBC using minimum height (0.3 m grid size). (Online version in colour.)

The results of this study are described below and summarized in table 2. In general, volume estimates were highest for each object using the Bounding Cuboid method, followed in descending order by the 3D CHP method, then the OHM, with SBC producing the lowest volume estimates. For example, the saltmarsh creek had an estimated volume of 1445.88 m^3^ by Bounding Cuboid, 881.89 m^3^ with 3D CHP, 769.66 m^3^ with OHM (0.5 m bin height) and 631.81 m^3^ with SBC. Voxel estimates sometimes resided between OHM and SBC estimates, as in *Ceiba pentandra* Tree 1 (27.11 m^3^ with 0.1 m voxel size) and *Ceiba pentandra* Tree 2 (52.92 m^3^ with 0.3 m voxel size) (figure 3). However, some voxel sizes produced estimates of volume exceeding even the 3D CHP, as in *Ceiba pentandra* Tree 3 (50 m^3^ with 1 m voxel size, 45.14 m^3^ with 3D CHP).

**Figure 4.**
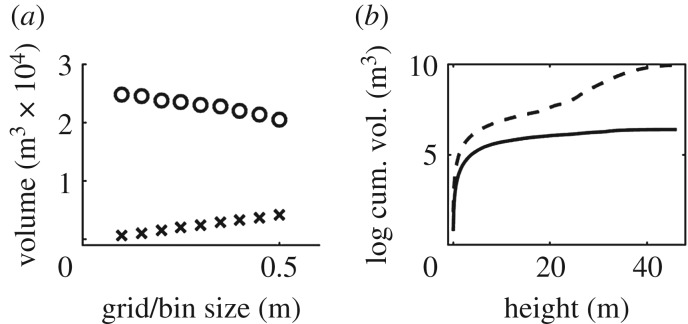
Volume estimation for *Ficus aurea* (*a*) by voxels (crosses) and Outer Hull Model (OHM, circles). Cumulative volume estimates (log scale) for the *Ficus aurea* over its height (*b*) according to voxels (solid line) and OHM (dashed line).

**Figure 5.**
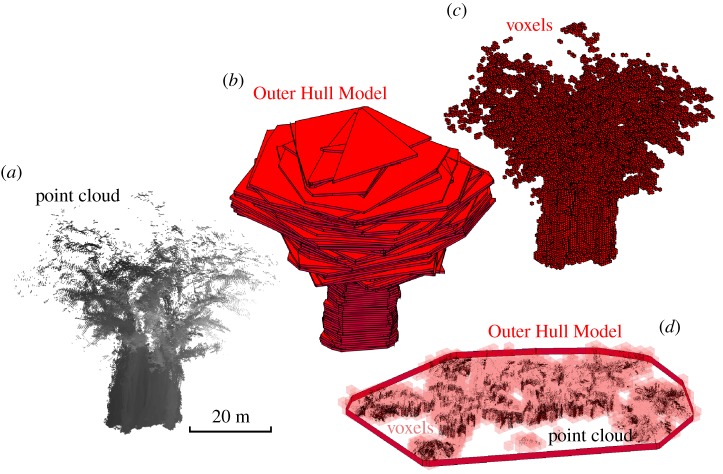
(*a*) Point cloud for *Ficus aurea*, (*b*) Outer Hull Model (OHM, 0.5 m bin height), (*c*) voxels (0.5 m voxel size) and (*d*) the combined point cloud, OHM and voxels visualization (bottom right, 2.5–3 m height) shows the relative operations of the methods in capturing the structure of the *Ficus aurea*. (Online version in colour.)

**Figure 6.**
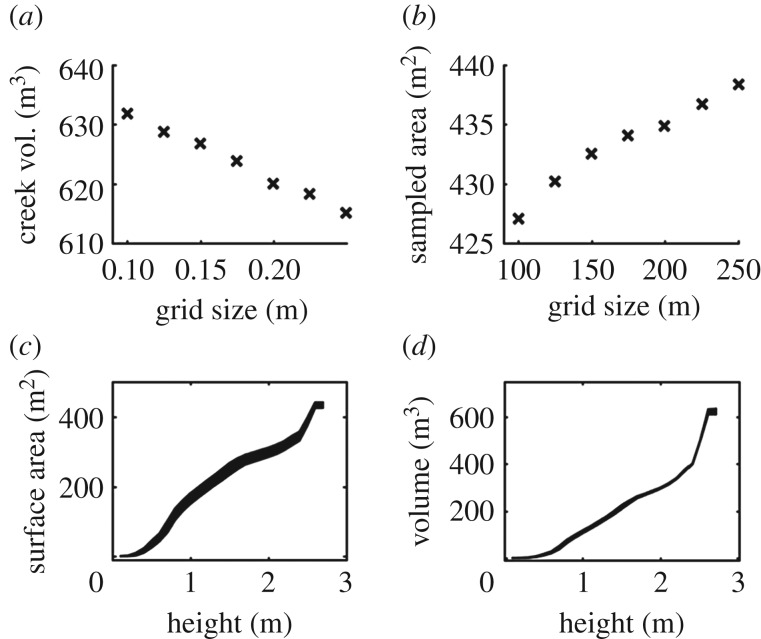
Volume estimation, and the area sampled with columns by the Square Based Column (SBC) method for the creek (*a*,*b*). Note the steady change in the volume estimation with the grid size. The surface area and volume estimations over height of creek reflect the profile of its structure (*c*,*d*). The thickness of the plot line represents 2 s.d. of variation about the mean.

**Figure 7.**
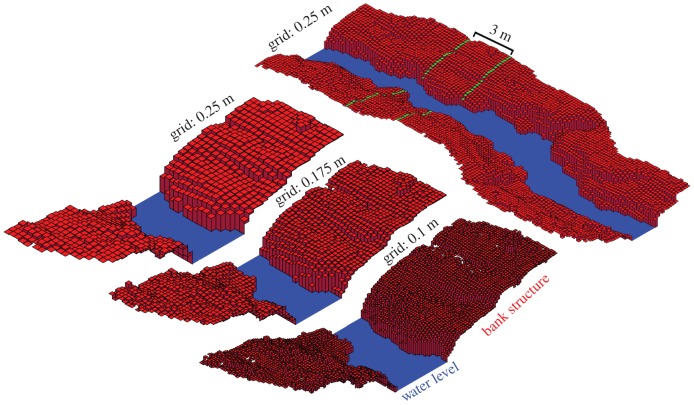
The Square Based Column (SBC) model of the saltmarsh creek in its entirety (top right) with 0.5 m grid size. Blue cells represent water level inferred from absence of lidar returns. Bottom left to right: Subsection of creek (marked by green lines) modelled with SBC (0.5 m, 0.25 m, 0.1 m grid sizes). (Online version in colour.)

**Figure 8.**
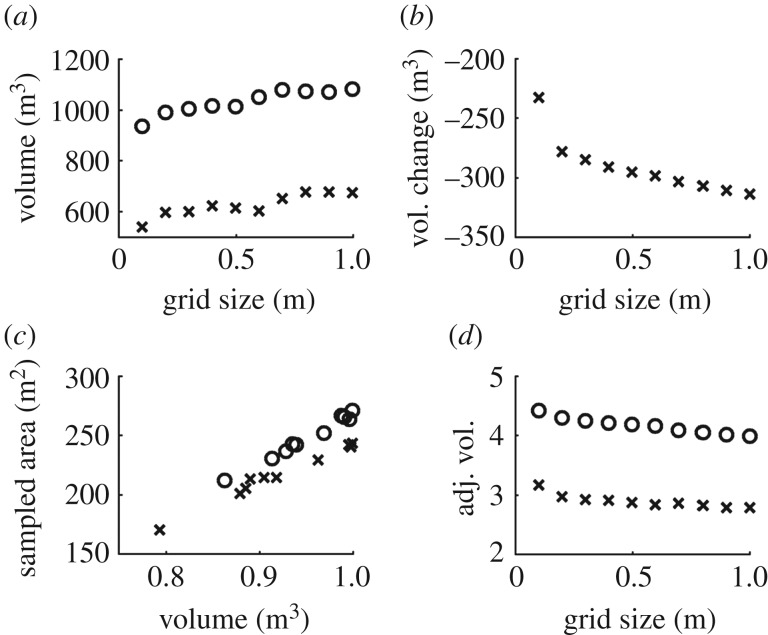
Volume estimation from the Square Based Column (SBC) method, for the eroding drumlin in 2014 (crosses) and 2015 (circles) with variation of SBC grid size shows estimated volume declining with grid size, which is counterintuitive (*a,b*). (*c*,*d*) The relationship between the sampled area and volume estimation for the eroding drumlin in 2014 (crosses) and 2015 (circles), and volume estimation for drumlin normalized by sampled area.

**Figure 9.**
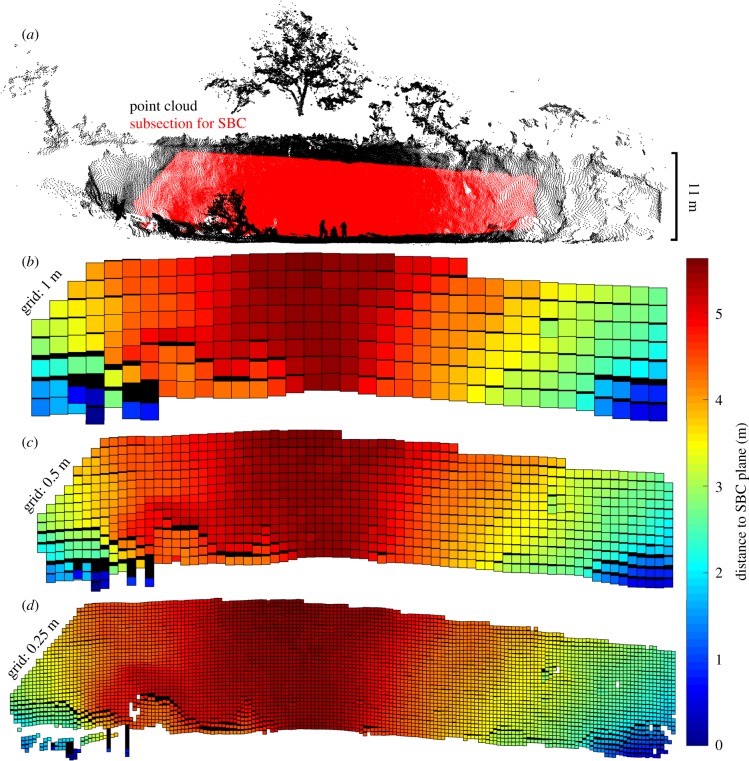
The point cloud for drumlin in 2015 (*a*) with the subsection for analysis displayed in red. Lidar operators, fallen tree and clifftop vegetation are visible. Square Based Column (SBC) models for 1 m (*b*), 0.5 m (*c*) and 0.25 m (*d*) grid sizes are visualized, coloured by distance to SBC plane. (Online version in colour.)

Parametrization produced a consistent response in volume estimations for OHM, with smaller bin heights producing higher estimates of volume in all cases. For example, *Ceiba pentandra* Tree 3 was estimated to be 31.34 m^3^ with 0.1 m bin height, 26.65 m^3^ with 0.3 m bin height, and 20.10 m^3^ with 0.5 m bin height (figures 4 and 5). The response of volume estimations with SBC was consistent in most cases, with smaller grid sizes producing higher estimates of volume. For example, the saltmarsh creek was estimated to be 631.81 m^3^ with 0.5 m grid size, 620.01 m^3^ with 0.3 m grid size and 615.08 m^3^ with 0.05 m grid size (figures 6 and 7). However, for the eroding drumlin, estimates of the volume decreased at smaller grid sizes (figure 8). This counterintuitive tendency was found to be closely related to the equivalent area sampled by the SBC at the various grid sizes. At smaller grid sizes, the resolution of the grid exceeded the resolution of the TLS sampling of the surface of the bluff, resulting in a proliferation of gaps evident in the reduction of sampled area (figures 8 and 9). Adjusting the volume estimates according to the area sampled restored the intuitive increase in volume with smaller grid sizes in both the coarser CBL1 (2014) and the finer CBL2 (2015) datasets (figure 8).

For the *Ficus aurea*, estimates of volume derived from OHM far exceeded those from voxels, regardless of parametrization of the two methods (24693.43–20452.94 m^3^ with OHM; 4087.88–581.06 m^3^ with voxels). The disparity between the methods' estimates was found to have a relationship to height. The lower regions of the *Ficus aurea*, comprising many, tightly packed vertical stems, had more closely related OHM and voxel estimates of volume. However, in the higher, canopy regions, estimates of volume with OHM were much larger than with voxels, reflecting the more diffuse branching structure of the tree, with less volume of vegetation spread over a much larger volume of space.

## 4. Discussion

In this study, we analysed estimates of volume for objects of ecological interest, including trees of various morphologies, a section of saltmarsh creek and a bluff on an eroding drumlin. The consistent relationships between the volume estimates of the various methods (Bounding Cuboid highest, then 3D CHP, then OHM and SBC lowest) was expected, given their intuitive interactions with object geometry. Bounding Cuboids and 3D CHP, by definition, encompass entire objects, characterizing the geometry of the space they occupy, rather than the geometry of the objects themselves. On the other hand, OHM and SBC provide geometric representations that conform more closely to the shape of the object. These principles also apply, at a finer structural scale, to explain the consistently higher volume estimations of OHM than SBC. OHM conservatively bounds the space occupied by each section of an object, while SBC allows the representation of more subtle geometric variation, particularly for undulating surfaces such as the saltmarsh creek (figure 7).

### 4.1. Bounding uncertainty in volume estimations

The predictable interactions of some of the geometric methods employed in this study with the geometry of the objects of ecological interest was leveraged to try and provide bounds on the uncertainty for the volume estimations of the various objects. Because the interactions of the methods with object geometry produce a consistent direction of bias, combining estimates of volume from multiple methods can help constrain the uncertainty in the true object volume. For example, 3D CHP will always overestimate object volume because it is limited to describing the largest spatial dimensions of the object. Conversely, SBC fitted to the interior of an object should always underestimate the volume because it will not integrate beyond the nearest component of the surface to the SBC plane.

**Figure 10.**
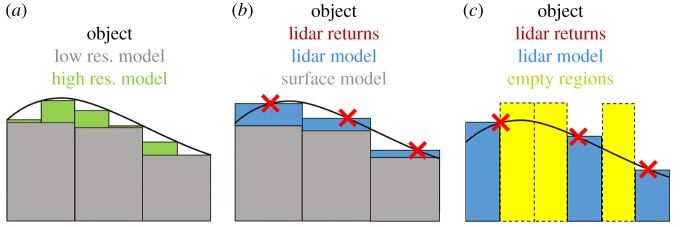
(*a*) The interaction of a geometric model with object geometry (black line) as parametrization changes. Grey rectangles show lower resolution geometric model, with green rectangles showing higher resolution. (*b*) Red crosses show the discontinuous sampling of lidar returns, with estimates based on lidar observations shown as blue rectangles. (*c*) Yellow rectangles show missed portions of object volume.

Figure 10 provides a demonstration of the interaction of a geometric model with object geometry as the parametrization of the model changes (figure 10*a*). As the model increases in resolution (from grey to green), the fit to the surface of the object (black line) becomes closer, including more of the volume of the object. However, as lidar data represent the surfaces of objects only discontinuously (red crosses as lidar returns, figure 10*b*), estimates based on lidar observations (blue rectangles) may not be constrained by the true bounds of the object (black line). Additionally, if parametrization of a geometric model leads to the resolution being too fine, relative to the observation density of the lidar, then portions of the structure may also be missed entirely (yellow rectangles, figure 10*c*).

**Figure 11.**
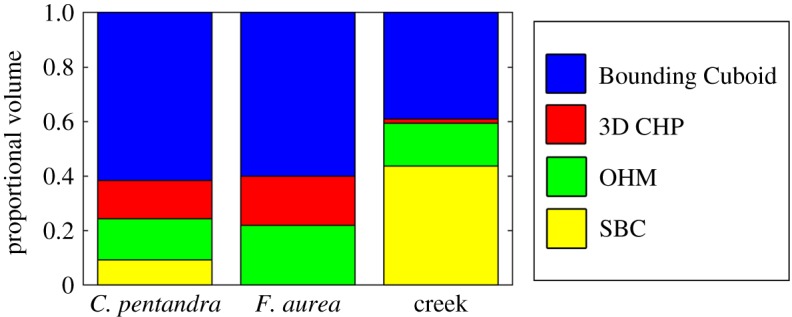
Comparison of the volume estimation methods for *Ceiba pentandra* 1, the *Ficus aurea* and the saltmarsh creek. Volume is expressed proportional to that of the Bounding Cuboid.

**Figure 12.**
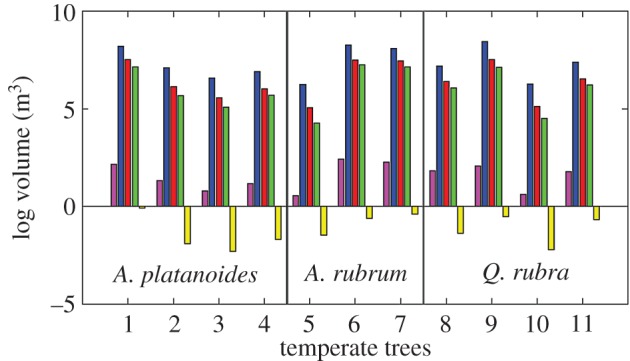
Comparison of the mean volume estimates from cylinder reconstructions [25], and volume estimates from other methods for the five *Acer platanoides*, three *Acer rubrum* and three *Quercus rubra* trees.

The potential to constrain uncertainty can be seen working effectively for the *Ceiba pentandra* tree bases (figure 3) and the saltmarsh creek (figure 7 and table 3), where the possibility space, as described by the proportion of the Bounding Cuboid, is substantially reduced in each case (figure 11). In the examples shown in figure 11, the green area represents the range between the maximum SBC estimate (where applied), and the minimum OHM estimate, and therefore should contain the true value in each case. However, the geometric complexity of complete trees, including the twelve temperate trees, and the *Ficus aurea* considered in this study, resists meaningful constraint of the possibility space for volume (figures 2, 5, 12, table 3). In figure 12, the green and yellow areas, which represent the range between the maximum SBC estimate, and the minimum OHM estimate, should contain the true value for tree volume. In fact, the volume estimates from the overestimating methods are orders-of-magnitude higher than the cylinder-based quantitative structure model (figure 12, table 3; see electronic supplementary material). This is unsurprising, because trees are the quintessential geometrically complex object, occupying far more space than they comprise volume.

**Table 3.** Volume estimates from the three-dimensional convex hull polygons (3D CHP), Outer Hull Model (OHM), voxels, Square Based Column (SBC) and cylinder reconstruction methods, expressed as the proportion of the volume estimate from the Bounding Cuboid method for each object. Three of the 12 temperate trees are included.

method of volume estimation	object							
	*C. pentandra* 1	*C. pentandra* 2	*C. pentandra* 3	*F. aurea*	saltmarsh creek	*A. platanoides*	*A. rubrum*	*Q. rubra*
3D CHP	0.38	0.25	0.27	0.39	0.61	0.51	0.46	0.39
OHM (min.)	0.22	0.08	0.12	0.18	0.53	0.35	0.37	0.27
SBC (max.)	0.09	0.05	0.47	—	0.44	2.57 × 10^−4^	1.39 × 10^−4^	1.27 × 10^−4^
cylinder reconstruction	—	—	—	—	—	2.43 × 10^−3^	2.93 × 10^−3^	1.71 × 10^−3^

Methods such as SBC and OHM cannot effectively characterize the internal structure of tree canopies, as they assume consistent relationships between the structure of objects and their surroundings, for example, that a saltmarsh creek is concave relative to a flat plane across its top. However, beyond the main stem, the branches and leaves of trees are numerous, and highly variable in their directionality. The direction and shape of each component of a tree canopy are heavily dependent on the component to which it is connected, and it is this principle that is leveraged by [25] in a quantitative characterization of trees as connected cylinders, existing in a hierarchical network.

### 4.2. Utility of geometric models

SBC showed the ability to represent both large and continuous forms of structure, such as the bluff, and structure which is wide-spread but consists of discrete components, such as the roots of the *Ceiba pentandra* (figure 3). Voxels can also achieve this distinction and can do so even when the components of structure have a complex three-dimensional spatial relationship, as in the *Ficus aurea* (figure 5), as opposed to the *Ceiba pentandra* tree roots (figure 3), which are primarily arrayed on the plane of the ground. However, there are several applications and scenarios in which SBC could potentially provide a more informative representation of structure than voxels. For example, lidar is limited to line-of-sight, and therefore will not produce any returns to mark the interior of objects. Therefore, the interior volume of objects will not be represented in voxels, but can be characterized with SBC, provided a suitable grid plane is employed (figure 3).

The methods examined in this study also show the potential to extract additional metrics of ecological interest. For example, SBC also provided the opportunity to retrieve metrics relevant to the hydrology of the saltmarsh creek from its structure. Estimates of water volume and water surface area for different water levels, representing the typical tidal range of the Plum Island saltmarsh were estimated directly from the SBC geometric model (figures 6 and 7). These metrics describe the influence of the structure of the creek on its hydrological function, showing the distinctly nonlinear surface area and volume gain with water level.

The SBC representations of *Ceiba pentandra* tree bases (figure 3) are a good example of how geometric methods can be complementary not just in their comparison (as when constraining uncertainty in volume estimations), but also in their combination. For example, SBC could conceivably be used to characterize the volume of buttressed roots, to supplement the characterization of the rest of a tree with the cylinder-based model. Indeed, when SBC was applied to the stems of the temperate trees in this study, it provided at least some lower bounds for volume estimates (table 3). Additionally, [27] has shown the OHM functioning to characterize tree stem taper.

We can also see the potential for hybridized geometric approaches in the structural characterization of the *Ficus aurea* by OHM and voxels (figures 4 and 5). The lower region of the *Ficus aurea* comprises many stem-like tap-roots creating empty space within the region typically assumed to be solid stem. OHM provides a representation of the overall superstructure of the tree, while voxels reveal the internal structure. Examining the volume estimates of the two methods as a function of height demonstrated how the voxel method discounts the empty regions that are included in the volume estimates of OHM (figure 5). The canopy of the *Ficus aurea* has a much higher ratio of overall extent to the volume of vegetation, and this is reflected by the much larger disparity in volume estimation higher up the tree, with a distinct elbow point at the transition from stem to canopy (figures 4 and 5).

### 4.3. Model response to parametrization can inform data quality evaluations

Under ideal data quality conditions, models such as SBC and OHM should have a response to their parametrization that is predictable, at least in terms of directionality. The smaller the grid size for SBC, for example, the more closely it can conform the structure of an object, the more volume it will integrate (figure 10). Likewise, the larger the bin height for OHM, the more likely it will be forced to encompass wider-spread points in each convex hull polygons, and therefore the higher the resulting volume estimations.

In most cases in this study, we see these relationships between model operations, object geometry and parameter variation emerging as expected in the results of this study (figures 4, 6 and 8, table 2). For the saltmarsh creek, *Ficus aurea* and *Ceiba pentandra* trees, volume estimates increased almost monotonically with reductions in SBC grid size and OHM bin height (figures 4, 6 and 8). As already discussed, the expectation of these relationships is based on assumptions about the geometry of the object (its shape and orientation within its surroundings). However, these relationships also rely on the assumed geometric tendencies of the object being preserved in the representation of the object by the TLS point cloud. If the TLS observations are not of sufficient resolution, or contain too much uncertainty from sources such as beam divergence and co-alignment error, then geometric models may no longer perform predictably (figure 8).

We see a clear example of this in the volume estimation of the eroding drumlin, where the volume estimation shows a positive correlation with the size of the SBC grid (figure 8), instead of the logical negative correlation. Examining the area sampled at each grid size revealed a steady decline in columns which found returns within their spatial bounds (figure 8). The amount of area sampled was strongly related to the resulting volume estimation, and dividing the volume estimation by the area sampled restored the logical relationship between volume estimation and SBC grid size (figure 8). While there is also change in the sampled area with changing grid size for the saltmarsh creek (figure 8), it is orders-of-magnitude less (8.9 × 10^−3^ coefficient of variation for creek area sampled, 0.1 coefficient of variation for eroding bluff). We can take away from this that the resolution of the TLS representation of the bluff is insufficient for characterization with smaller grid sizes of SBC. The anomalously small estimated change in volume between 2014 and 2015 at 0.1 m grid size (figure 8) could suggest that SBC grid size should be at least greater than 0.1 m for this dataset.

This example demonstrates how the predictability of the bias and response of certain geometric methods can act as an indicator of the quality of underlying data, in terms of its representation of an object of ecological interest. Methods such as SBC and OHM can provide a warning of, at least, serious departures from assumptions about data quality, and at most guide their own, and other geometric models' parametrizations through examination of their responses. All geometric methods will have tendencies, such as the tendency for voxel estimates of volume to increase with an increase in voxel size, because the presence of discrete points is always being extrapolated into a minimum amount of volume. However, departures from these tendencies are just as likely to be a function of the geometry of the object under scrutiny, as they are to be indicative of a data quality issue.

### 4.4. Boundary effects in models that partition space

It is important to note that all geometric methods that employ spatial partitioning of a point cloud (including OHM, SBC and voxels) can be influenced by boundary effects when their spatial parameters are changed. Boundary effects, in this case, refer to the change in membership of a set of points within a partition due to the movement of the boundaries of the spatial partition. The aggregation of the different set of points holds the potential for creating unexpected responses in volume estimates. For example, the geometry of the saltmarsh creek consists of slopes interspersed with plateaus (figure 7), which could result in drastic boundary effects as each plateau is included within different, or different numbers of height bins. However, even with the pronounced geometry of the creek, there is little evidence of boundary effects in the example in this study, beyond a slight unevenness in the rate of volume estimate change with SBC grid size (figure 6). Furthermore, when extrapolated to useful hydrological modelling products such as water volume and surface area over height (figure 6), SBC grid size causes very little variation.

### 4.5. Terrestrial laser scanner data quality and processing can violate assumptions of uncertainty bounds

Evidence of the interactions between sampling density of TLS observations and geometric models has been described above, but sampling density warrants direct consideration. Some geometric techniques, such as cylinder-based reconstruction, can be sensitive to uneven point densities, requiring normalization of point densities. Most of the other geometric methods included in this study are most vulnerable to the minimum point density in a TLS observation, rather than the evenness of the point density, hence the lack of normalization of point density in the TLS data used. Additionally, the technology is advancing so rapidly, in terms of resolution and accuracy, that many TLS instruments may be producing representations of objects that are functionally continuous, such that they would exhibit the same behaviour when interacting with simple geometric models as the true object. However, this is certainly not true of the instrument used in this study, and an evaluation of the required point densities would be required to make effective retrievals for larger scale studies.

Each lidar return can have error in its range, and therefore its position in a point cloud, as well as errors resulting from the interaction of lidar pulses with object geometry. These sources of error in the position of points can have serious implications with the simpler geometric models. Methods that are controlled by the extremities of an object, such as 3D CHP, will essentially respond to the largest positional error in the direction of its bias, because that positional error will form the boundary of the resulting polygon. The most extreme case is the Bounding Cuboid, which is ultimately controlled by a single pair of points in each spatial axis, and therefore a small deviation of any of those controlling points in the TLS data propagates to a large increase in volume estimation.

A more fundamental challenge to the methods in this study, and to TLS modelling of ecologically important objects in general, is that TLS data do not represent objects continuously, accurately or completely. Instead, we have discrete and uncertain observations of an object, which vary in density. In terms of this study, this means that the assumptions concerning interactions between methods and object geometry may be violated. As discussed, this can result in unexpected relationships between methods and responses of methods to parametrization, and also assumptions of bias in the estimation of geometric properties.

One potential response to a TLS observation quality failing to meet the demands of a geometric model is to employ interpolation and smoothing techniques to address density and accuracy issues, respectively. While these are intuitively appealing approaches it should be noted that their use would immediately invalidate the assumptions of bias, as elements of object structure are being established or modified based on association, rather than by direct observation. Furthermore, there are substantial challenges to appropriately weighting interpolation, particularly for geometrically complex objects, and to determine the regions of the object to which such an interpolation should be applied.

### 4.6. Representativeness of results

This study considered multiple volume estimation methods and several distinctly different forms of ecologically relevant objects. However, the sample size for each method and object only qualify as initial demonstrations and examples. Given that a major consideration of the paper are the unique challenges of esoteric object forms, it is important to note that there are many ecological objects, even from the same categories and ecosystems featured in this study, that will provide significant hurdles to application of the methods in their current form. For example, *Ficus aurea* can have extremely variable morphological arrangements that may violate the assumptions of volumetric models, and the individual in this study was free-standing, while other examples may still be structurally entwined with the host trees. Another example would be that the geomorphology of saltmarsh creeks, even within the same marsh as the sampled creek, has been observed to be breaking down over time, losing the consistent plateaus and potentially challenging the relative success of the methods seen in the limited example herein.

Additionally, many of these datasets were acquisitions of opportunity, targeting the most accessible examples of a type of ecological object, or the most ideally situated object within an ecosystem. To give some examples, the *Ceiba pentandra* trees selected were those least obscured by understorey foliage, and the temperate trees were all in open urban settings without any canopy overlap. Such examples demonstrate that there remain considerable challenges of characterizing ecosystems, which are also well documented elsewhere [35–37]. However, part of the reason that so many challenges have been identified is because the continuing development of lidar technology, TLS instruments in particular, and lidar data processing and analysis is encouraging sampling in more inhospitable and remote ecosystems, comprehensive and higher quality acquisitions within ecosystems, and sampling under less favourable conditions. Therefore, while this study should not be treated as validation of the operational readiness of any particular method or instrument in any particular ecosystem, it is an effort towards repurposing and expanding the tools of geometric modelling to meet some of the existing and forthcoming challenges of TLS sampling of ecosystems.

## 5. Conclusion and further implications

The most prominent ecological applications of TLS data, such as the estimation of tree volume, will typically give rise to standardized geometric modelling methods, such as the cylinder-based quantitative structure model described in this study [25]. While such models are likely to provide the most accurate estimates of geometric properties, the uncertainty in those estimations can only be calculated as error, established with direct validation. Since there is no guarantee that the true value of a geometric property lies within the bounds of error in a downstream product, this study investigated whether geometric models with known directions of bias could provide uncertainty bounds to supplement methods which more closely conform to object geometry.

It was found that in less structurally complex objects such as saltmarsh creeks, and even in the less structurally complex regions of trees, such as the bases of *Ceiba pentandra*, well-suited geometric models could provide meaningful constraints to object volumes. However, where models were less well suited to the morphology of an object, they did little to constrain the uncertainty in volume estimates. For trees canopies, which have a complex structural relationship with the overall space they occupy, none of the coarser geometric techniques investigated in this study could provide meaningful bounds of uncertainty for the estimates of volume from cylinder-based reconstructions.

Since many of the geometric properties of objects that we seek to observe with TLS become inputs into larger area ecological modelling efforts, constraining the uncertainty for these inputs is important. Along with the potential of validating coarser resolution satellite estimates of ecosystem properties, especially relevant to the forthcoming GEDI mission [31], these valuable potentially applications recommend that work continues to provide constraints for TLS observations of even structurally complex objects such as trees.

There may be potential for constraining the bounds of uncertainty further in hybridized models that use more optimal modelling techniques for different components of an object, such as the stem and canopy of a tree, while maintaining a consistent direction of bias between the models. Some of the models described here may also directly augment cylinder-based reconstructions. For example, the quantitative structure model of [25] could be used to model the upper stem and branching structure of tropical trees as a hierarchical network of cylinders, supplemented by SBC representations of the buttress root structure.

OHM and SBC, as emerging methods, showed the potential to be used to produce independent characterizations of objects of appropriate geometry. In particular, the representations of the unique morphologies of tropical vegetation, such as the buttressed roots of *Ceiba pentandra* trees and the complex superstructure of *Ficus aurea*, encourage further application of the methods. These methods may also provide further technical benefits, such as the format of SBC providing an intuitive pathway for reducing the computational intensity and storage requirements of voxel datasets.

As the cylinder-based tree modelling methods are already being combined with stem identification techniques to move to stand-level operation [26,38], the geometric models described here could actually be used to classify the presence of morphological features such as buttress roots (for example, by detecting stem diameter changes with OHM), as well as then providing the necessary hybridized modelling. In general, as ecological assessment with lidar instruments continues to expand, adding to the toolbox of geometric models available to characterize fringe, outlier and novel data scenarios should prove useful.

Hybridizing geometric models may also provide novel ways to describe the geometry of objects. For example, there will be more disparity between 3D CHP and Bounding Cuboid volume estimates when there is more variation in the dimensions of different regions of the object. In other words, objects with more morphological heterogeneity will have higher ratios of Bounding Cuboid to 3D CHP volume than more homogeneously structured objects. Similarly, strong disagreements between volume estimates from 3D CHP and OHM suggest there is a wide range of spatial extents across regions of an object. This concept is easily visualized for trees, where the disparity between the extent of tree crowns and trunks would be reflected in a large difference in volume estimates between 3D CHP and OHM. Investigating the characterization of object geometry according to their relative representation by geometric models may have particular application to airborne lidar observations, which tend to be coarser but have larger spatial extents, and therefore sample sizes for objects of ecological interest.

This study primarily discussed ecological geometric modelling with volume as the response variable. While volume may be a product of direct ecological interest in some cases, such as for biomass and timber yield estimations in forestry, work to examine other fundamental geometric products, such as surface area [39–41], will be highly beneficial. Also warranting consideration is the transfer of the findings of this study from the upstream geometric modelling to additional, downstream ecological modelling applications, including hydrological properties of interest (water volume and surface area as a function of tidal height) and geological properties of interest (erosion of material over time).

Ultimately, the continuing expansion of TLS applications will continue to bring established geometric modelling methods into contact with novel, non-ideal and outlier cases for which they may not have been designed, or to which they may be unpredictably sensitive. The development and understanding of diverse geometric modelling tools, as this study sought to further, will best serve to adapt the most refined models to new conditions. This is especially true for forestry applications, as improvements in the capabilities, qualities and practicalities of TLS instruments are encouraging studies in ecosystems such as tropical forest which are morphologically unusual and inaccessible, but increasingly essential to characterize for management and global modelling purposes.

## Supplementary Material

Full Cylinder Model Results

## Data accessibility

Terrestrial laser scanner data, Matlab scripts and cylinder-based quantitative structure model results relevant to this paper are available via FTP at: ftp://rsftp.eeos.umb.edu/data02/OpenAccessPaperData/RSCode_Materials.zip.
