# Supplementary material for: Bounding uncertainty in volumetric geometric models for terrestrial lidar observations of ecosystems
Source: Interface Focus. 2018 Feb 16;8(2):20170043. doi: 10.1098/rsfs.2017.0043 (PMC5829184; doi:10.1098/rsfs.2017.0043)
Supplement: Full Cylinder Model Results [file rsfs20170043supp1.pdf]

Supplementary Table: Cylinder based quantitative structure model results for temperate trees.

	Method of Volume Estimation					
**Tree Species**	**QSM Mean (m^3)**	**QSM Min (m^3)**	**QSM Max (m^3)**	**3D CHP (m^3)**	**2D SCHP (m^3)**	**Trunk SBC (m^3)**
**Norway Maple**	8.760	7.306	9.708	1846.8	1273.3 - 1336.7	0.924 - 0.975
**Norway Maple**	3.766	2.984	4.117	457.48	289.82 - 309.16	0.149 - 0.207
**Norway Maple**	2.213	1.901	2.460	258.873	160.25 - 172.42	0.101 - 0.156
**Norway Maple**	3.260	2.589	3.972	417.50	296.77 - 313.54	0.184 - 0.253
**Norway Maple**	1.738	1.459	2.040	155.92	71.137 - 77.108	0.232 - 0.245
**Red Maple**	11.400	8.783	12.550	1787.344	1423.6 - 1450.6	0.543 - 0.648
**Red Maple**	9.708	8.743	10.628	1740.483	1273.5 - 1296.7	0.673 - 0.736
**Red Maple**	6.196	5.014	7.284	594.669	433.54 - 458.62	0.255 - 0.313
**Red Oak**	7.995	7.821	8.169	1846.689	1241.6 - 1309.6	0.591 - 0.675
**Red Oak**	1.873	1.187	2.451	168.302	90.472 - 99.742	0.110 - 0.131
**Red Oak**	6.003	3.896	7.048	690.290	499.86 - 525.10	0.507 - 0.535
